# An Aptasensor Based on a Flexible Screen-Printed Silver Electrode for the Rapid Detection of Chlorpyrifos

**DOI:** 10.3390/s22072754

**Published:** 2022-04-02

**Authors:** A. K. M. Sarwar Inam, Martina Aurora Costa Angeli, Ali Douaki, Bajramshahe Shkodra, Paolo Lugli, Luisa Petti

**Affiliations:** 1 Sensing Technologies Laboratory, Faculty of Science and Technology, Free University of Bozen-Bolzano, 39100 Bolzano, Italy; akminam@unibz.it (A.K.M.S.I.); ali.douaki@unibz.it (A.D.); bajramshahe.shkodra@unibz.it (B.S.); paolo.lugli@unibz.it (P.L.); luisa.petti@unibz.it (L.P.); 2 Department of Nutrition and Food Engineering, Daffodil International University, Dhaka 1207, Bangladesh

**Keywords:** aptasensor, electrochemical sensor, organophosphorus pesticide, chlorpyrifos, flexible substrate, screen-printed sensor

## Abstract

In this work, we propose a novel disposable flexible and screen-printed electrochemical aptamer-based sensor (aptasensor) for the rapid detection of chlorpyrifos (CPF). To optimize the process, various characterization procedures were employed, including Fourier transform infrared spectroscopy (FT-IR), electrochemical impedance spectroscopy (EIS), and cyclic voltammetry (CV). Initially, the aptasensor was optimized in terms of electrolyte pH, aptamer concentration, and incubation time for chlorpyrifos. Under optimal conditions, the aptasensor showed a wide linear range from 1 to 10^5^ ng/mL with a calculated limit of detection as low as 0.097 ng/mL and sensitivity of 600.9 µA/ng. Additionally, the selectivity of the aptasensor was assessed by identifying any interference from other pesticides, which were found to be negligible (with a maximum standard deviation of 0.31 mA). Further, the stability of the sample was assessed over time, where the reported device showed high stability over a period of two weeks at 4 °C. As the last step, the ability of the aptasensor to detect chlorpyrifos in actual samples was evaluated by testing it on banana and grape extracts. As a result, the device demonstrated sufficient recovery rates, which indicate that it can find application in the food industry.

## 1. Introduction

Organophosphorus pesticides are among the most widely used compounds to control pests and diseases in the agricultural sector of developing countries [1]. Chlorpyrifos (CPF), one of the most widely used organophosphorus pesticides in the international market, is a major concern because of its toxicity, which leads to food safety problems [2]. The CPF is designed to kill pests by blocking the acetylcholine esterase, an enzyme that allows synaptic transmission [3]. In the same manner, it is harmful to humans, causing acute neurological toxicity, neurological disorders, and reproductive disorders [4,5]. Exposure to CPF can also generate oxidative stress and DNA damage [6]. Because of the hazardous effect of CPF on human health, the World Health Organization (WHO) and European directives have set the acceptable daily intake of CPF as 30 and 0.1 ng/mL in drinking water, respectively [7,8], while some developed countries have started to ban this for agricultural purposes. However, CPF residues are still present in soil and food products [9]. It is therefore paramount to be able to detect the presence of CPF in agriculturally derived food products such as cereals, fruits, and vegetables [10].

Until now, monitoring the level of chlorpyrifos residue in food has been mainly performed exploiting different conventional analytical techniques such as high-performance liquid chromatography (HPLC), gas chromatography (GC) coupled with nitrogen phosphorus detector (GC-NPD), and mass spectrometry (MS) [11,12,13]. Despite the high sensitivity and low detection limit of these instrument-based techniques, developing and underdeveloped countries, which still use CPF in agriculture, need to have cost-effective and simpler alternatives for food authentication and monitoring. An interesting solution is offered by low-cost biosensors, which enable quick, cost-effective, on-site assessments that do not require trained personnel [13,14,15,16].

Several biosensors characterized by different transducing mechanisms, for example, electrochemical, optical, or mass-based, have been applied for the detection of CPF during the last decades [14,17,18]. Among all, the electrochemical method has recently become an attractive choice because of its quick and highly sensitive response, coupled with the ability to integrate with a wide range of biorecognition elements such as aptamers, enzymes, or antibodies easily [19]. Compared to other biorecognition elements, aptamers can be integrated with an electrochemical sensor providing high sensitivity and selectivity with easily detectable signals [20]. Aptamers are synthetic single-stranded RNA or DNA molecules having a specific 3D structure that is chosen by random oligonucleotide libraries using an in vitro selection process called systematic evolution of ligands by exponential enrichment (SELEX) [21]. Aptamers show distinct advantages if compared to enzymes and antibodies, such as facile preparation, preservation, modification, and good thermal stability [22]. Different aptamer-based sensors (aptasensors) have been extensively designed using various functional materials such as gold nanoparticles (AuNPs), carbon nanotubes (CNTs), fullerene, exonuclease I, chitosan (Cs), etc., to electrochemically detect various trace analytes [23,24,25,26,27]. In recent years, few electrochemical aptasensors have been realized for the detection of CPF. An ultrasensitive aptasensor made of a glassy carbon electrode (GCE) modified with mesoporous carbon functionalized by chitosan and multiwalled carbon nanotubes (MWCNTs) reported a detection range between 1 and 10^5^ ng/mL and a limit of detection (LOD) of 0.33 ng/mL [28]. Similarly, Jiao et al. presented a GCE functionalized with chitosan, carbon black, and graphene oxide @Fe_3_O_4_ together to produce a complex immobilization matrix for aptamers [29]. Xu et al. [30] modified the GCE with carboxyl-functionalized single-walled carbon nanotubes (SWCNTs) and electrodeposited cuprous oxide (CuO) nanoflower, obtaining one of the lowest detection limits (0.07 ng/mL) if compared to the other aptasensors [4,17,19,31,32]. Even if the above-cited works showed outstanding performances in terms of sensitivity and LOD, they are made of complex fabrication procedures that involve the use of several materials, reducing the possibility of the scale-up.

To overcome these issues, the preparation of the CPF aptasensor presented in this study introduces a simple and cost-effective screen-printed silver electrode (SPAgE) on a flexible platform, leading at the same time to a lowering of the LOD with a wider detection range. To fulfill the aim of achieving a portable device to perform an in situ measurement, a screen-printed compact flexible electrochemical platform fabricated by Ag ink has been realized. Ag ink was chosen over gold because of its cost-effectiveness and over the well-studied carbon because of its higher conduction and faster electron transfer rate [33,34]. The screen printing technique provides several extra benefits, such as roll-to-roll large production, ability to withstand mechanical stress, easy implementation, and adaptability, if compared to other printing methods [35,36,37,38,39]. Moreover, a flexible and low-cost polymeric (polyethylene terephthalate (PET)) substrate was used, in order to pave the way to the use of scalable fabrication techniques and achieve advantages such as being unbreakable, light, and thin in weight, as well as the possibility to apply innovative ideas [40]. To the best of our knowledge, this is the first work reporting the functionalization of an aptasensor using a flexible SPAgE for the detection of CPF. The uniqueness of the proposed sensor is the fact that it is cost-effective and simple, with few fabrication steps if compared to the other sensors presented in the literature, without losses in performance. The proposed sensor showed a good capability of CPF detection with a low detection limit (0.097 ng/mL) and wide linear detection range (from 1 to 10^5^ ng/mL). The sensors also showed good selectivity versus the most common interfering agents (dichlorvos, malathion, carbofuran, deltamethrin, and metamitron). The sensor was also investigated for stability revealing a stable shelf life of two weeks. The proposed sensor was validated by testing CPF concentrations on banana and grape samples, showing a relative recovery of 97.7% to 105.7% and 103.5% to 104.1% and a coefficient of variance of 2.7% to 4.5% and 1.7% to 3.7%, respectively. In the broader spectrum of the food safety sector, this sensor showed significant potential in terms of high sensitivity and wide detection range by utilizing a cost-effective flexible platform and a facile screen-printing technique for the detection of CPF.

## 2. Materials and Methods

### 2.1. Chemicals and Apparatus

N(3-dimethylaminopropyl)N ethylcarbodiimide (EDC), N-Hydroxysulfosuccinimide sodium salt (NHS), 11-mercaptoundecanoic acid (11-MUA), phosphate buffer saline (PBS), potassium chloride (KCl), Isopropyl alcohol (IPA), potassium ferricyanide III (K_3_[Fe(CN)_6_]), potassium ferricyanide II trihydrate (K_4_[Fe(CN)_6_] × 3H_2_O) and pesticides (chlorpyrifos, carbofuran, dichlorvos, malathion, deltamethrin, and metamitron) were purchased from Merck KGaA, Darmstadt, Germany. The aptamers with an amine group at the 5′-end, selected from previously reported literature [41], were obtained from Biomers (Ulm, Germany). The sequence of chlorpyrifos oligonucleotides containing 91 bases is shown here: 5′-NH_2_-(CH_2_)_6_−CCTGCCACGCTCCGCAAGCTTAGGGTTACGCCTGCAGCGATTCTTGATCGCGCTGCTGGTAATCCTTCTTTAAGCTTGGCACCCGCATCGT-3. Then, 0.01 M PBS (pH 7.0) was used to prepare the different concentrations of CPF. PBS (0.01 M, pH 7.0) containing 5 mM [Fe(CN)_6_]^3−/4−^ and 0.1 M KCl was used as an electrolyte [42]. All chemicals used in this work were analytical grade and were used without any further purifications. Electrochemical measurements, for example, cyclic voltammetry (CV) and electrochemical impedance spectroscopy (EIS) were performed by using a potentiostat (VersaSTAT 4 workstation, Princeton Applied Research, Oak Ridge, TN, USA) in the air at room temperature. The Fourier-transform infrared (FT-IR) spectroscopy (INVENIO-R; Bruker, Karlsruhe, Germany) using a diamond crystal was performed for the characterization of the aptasensor.

### 2.2. Fabrication of Aptasensor

**Figure 1:**
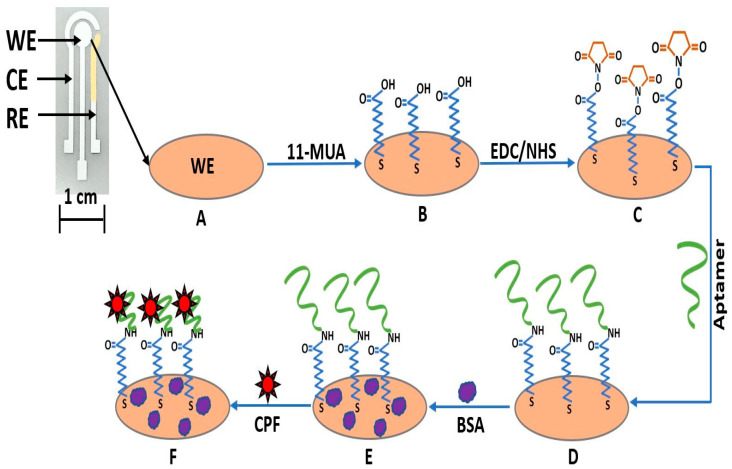
The step-by-step fabrication process and working mechanism of the printed aptasensor for chlorpyrifos detection. The sensor is made of a three-electrode system: working electrode (WE), counter electrode (CE), and reference electrode (RE). (**A**) Bare working Ag electrode (WE), (**B**) 11-MUA attached to the WE with thiol bond, (**C**) EDC/NHS immobilization with 11-MUA, (**D**) covalent bonding between the amine group of aptamers with the carboxylic group of 11-MUA, (**E**) BSA immobilized on top of the electrode to eliminate non-specific binding, and (**F**) CPF attached with aptamer.

The developed sensor employs a typical three-electrode electrochemical structure, printed on top of a 125 micron thickness PET foil (Rauch GmbH) substrate using a semi-automatic screen-printing machine (Aurel automation S.P.A. C290, Modigliana, Italy). Figure 1 shows the sensor structure consisting of an Ag (ECI 1011, LOCTITE E&C) working electrode (WE) (diameter: 4 mm), an Ag counter electrode (CE), and an Ag/AgCl (ECI 1011, LOCTITE E&C) pseudo-reference electrode (RE). The three-electrode structure of the sensor was fabricated as described by Inam et al. [43]. Subsequently, the electrodes were ultrasonically cleaned at room temperature in IPA and double-distilled water for 5 min using a bath sonicator (CP 104, Vetrotecnica S.r.l., Padova, Italy) [40].

The biofunctionalization of the WE as shown in Figure 1 was performed using 11-MUA, which is a binder molecule, characterized by a thiol group (-SH) at one end and a free carboxylic group (-COOH) on the other end. First, the electrode was immersed in 2 mM of 11-MUA for 24 h, promoting the formation of the self-assembled monolayer (SAM) [44]. Afterward, the electrodes were rinsed with DI (deionized) water to remove the excess of 11-MUA and air-dried at room temperature. The -SH group, due to the high affinity to conjugate with noble metals, attaches to the Ag working electrode forming the SAM, while the -COOH group remains free for subsequently amide coupling of the aptamers [45]. To form a strong amide bond, first, the activation of the -COOH group was performed via EDC/NHS chemistry. Therefore, 6 µL of 300 mM of EDC and 35 mM of NHS were dropcasted on top of WE and kept for 1 h as previously employed by Nerantzaki et al. [46]. Afterward, on top of each WE, 6 µL of 1 µM aptamer dispersion (diluted in double-distilled water) was dropcasted and kept at 4 °C for 16 h. The reaction of these chemicals with the -COOH leads to a semi-stable amine-reactive NHS-ester group, which once exposed to the aptamers, reacts with the primary amines (found in aptamers) to form a strong and stable amide bond [45]. The electrodes were rinsed with DI water to remove the excess aptamers and, finally, incubated with 0.5% of BSA to ensure specific binding. Due to this blocking step, the recorded signal can be ascribed only to the interaction between aptamers and the analyte [47]. Finally, the electrodes were rinsed with DI water and kept in the refrigerator at 4 °C when not in use.

### 2.3. Preparation of Fruit Samples

To validate the sensor in practical applications, a real sample evaluation was performed. For this experiment, fresh bananas and grapes were purchased from a local store. The samples were prepared according to the following procedure. First, 100 g of each fruit was crushed with a juicer and diluted in 100 mL of 0.01 M PBS. Then, the solution was sonicated for 5 min followed by 5 min centrifugation at 8000 rpm. Only the supernatant was collected for analysis. Before use, the supernatant was filtered through a 0.250 µm membrane. This resulting suspension was used for the preparation of CPF concentration as a part of the real sample analysis.

## 3. Results

### 3.1. Characterization of the Aptasensor

**Figure 2:**
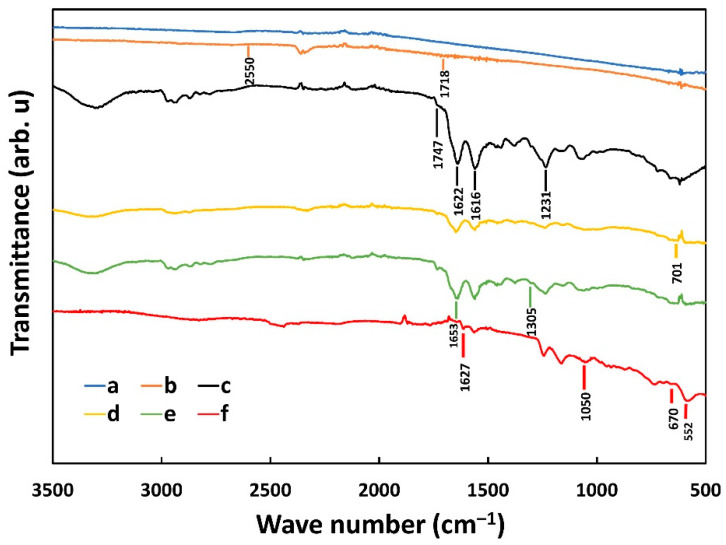
FT-IR spectra during step-by-step fabrication process of the aptasensor. Spectra of (a) Ag electrode, (b) 11MUA-Ag electrode, (c) EDC/NHS-11MUA-Ag electrode, (d) Aptamer-EDC/NHS-11MUA-Ag electrode, (e) BSA- Aptamer-EDC/NHS-11MUA-Ag electrode, and (f) Sensor after electrochemical measurements.

To characterize the biofunctionalization process used for the realization of the aptasensor, FT-IR was performed at each step. FT-IR allows for investigating the chemical bonding, hence indicating successful immobilization at each step. For the FT-IR analysis, transmittance mode was used to record the spectra over the 500–3500 cm^−1^ range with a resolution of 4 cm^−1^. Curves a and b in Figure 2 show the infrared spectrum of the Ag working electrode before and after the 11-MUA treatment, respectively. The successful bonding between 11-MUA on the Ag electrode is proven by the fact that the -SH group, which normally appears at 2550 cm^−1^, is absent in curve b [48,49], whereases the peak of the carboxyl group, which is free at the other end of 11-MUA, is visible at 1718 cm^−1^ [50]. After the incorporation of EDC/NHS, (Figure 2, curve c), a peak at 1747 cm^−1^ appears, representing the asymmetric carbonyl stretch ʋ(C=O) of the semi-stable amino reactive NHS ester group [51]. The presence of EDC/NHS is also proven by the appearance of a new peak at 1622 cm^−1^ (in curve c), which represents the amide (I) band (CO stretching around 80%, CN stretching around 10% and NH bending vibration around 10%). Moreover, curve c showed the amide (II) band at 1516 cm^−1^ (NH bending vibration around 60% and CN stretching around 40%) and amide (III) band at 1231 cm^−1^ (CN stretching 30%, NH bending vibration of 30%, CO stretching of 10%, O=C–N bending vibration 10%), confirming the presence of EDC/NHS [52]. The immobilization of aptamers, which has the -NH_2_ group at the end, is proven by the presence in curve d of the band at 701 cm^−1^, attributing to -NH_2_ stretching vibration [53]. Finally, in Figure 2, curve e, two bands at 1305 and 1653 cm^−1^ appeared due to the presence of BSA that have both amide (I) and amide (II) bonds (α-Helix) [54]. Furthermore, FT-IR was performed again after the electrochemical measurement of 100 ng/mL of CPF with the sensor to observe the morphological condition of a used sensor. The peak (curve f) at 552 cm^−1^ is due to the stretching of Fe-C≡N which proves the presence of [Fe(CN)_6_]^3−/4−^ [55]. Conversely, the peaks at 1627, 1050 and 670 cm^−1^ represent the C=O, C-O and C-Cl stretching, respectively, proving the presence of chlorpyrifos [56].

**Figure 3:**
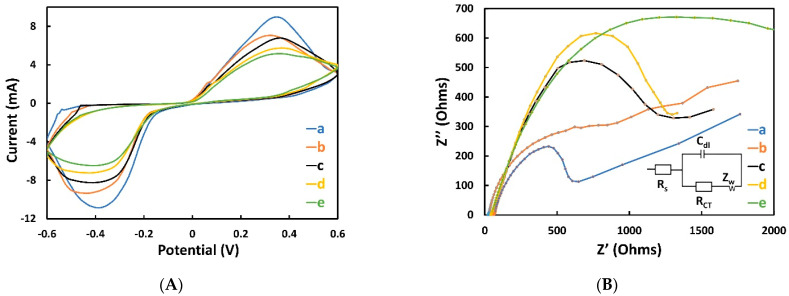
CV (**A**) and EIS (**B**) during the step-by-step fabrication process of the aptasensor. (a) Ag electrode, (b) 11MUA-Ag electrode, (c) EDC/NHS-11MUA-Ag electrode, (d) Aptamer-EDC/NHS-11MUA-Ag electrode, and (e) BSA-Aptamer-EDC/NHS-11MUA-Ag electrode. Here, 0.01 M PBS containing 5.0 mM [Fe(CN)_6_]^3−/4−^ and 0.1 M KCl was used as an electrolyte, and screen-printed Ag/AgCl was used as the RE.

Additionally, CV and EIS were used in every step of the fabrication process to investigate the changes in electron transfer and surface resistance of the electrode. For both measurements, three electrodes (WE, CE, and RE) were covered with 50 µL of the electrolyte solution (0.01 M PBS containing 5.0 mM [Fe(CN)_6_]^3−/4−^ and 0.1 M KCl). For CV measurements, the scan rate was set at 100 mV/s, and the sweeping potential range was set between −0.6 and 0.6 V. As shown in Figure 3A, a well-defined redox peak for every CV measurement at each characterization step is present. The CV of the bare Ag electrode (Figure 3A curve a) showed the highest reduction peak because of the high conductivity of Ag. After immobilizing 11-MUA, the redox peak decreased (curve b) significantly because it blocked the electron transfer [41]. After the incorporation of EDC/NHS (curve c), aptamer (curve d), and BSA (curve e), the redox peak amplitude decreased progressively because of their non-electrochemical activity that blocked the electron transfer between electrolyte and electrode, as also shown by Roushani et al. [41]. More specifically, DNA aptamers comprise phosphate groups and sugars, and the phosphate groups are negatively charged, thus giving the aptamers a negative charge. Consequently, the immobilized aptamers act as a barrier for electron transfer on the electrode surface, thus repelling the [Fe(CN)_6_]^3−/4−^ anions and reducing the generated current [57].

Another effective method for further characterization of an electrode’s surface feature is EIS, from which the Nyquist diagrams are derived [58]. To explain the impedance output and to relate the biological to the electrical domain, the Randles equivalent circuit (inset of Figure 3B) was used. According to this model, the capacitance between the electrolyte and the electrode is represented by C_dl_. In addition, to model the diffusion process of the anions in the electrolyte (bulk) toward the electrode surface, the Warburg element (Z_w_) is used. The electrolyte resistance is represented by R_s_, which was almost constant (23–46 Ω), due to the use of the same electrolyte during the EIS experiments. The electron transfer between an electrolyte and an electrode surface is denoted by R_CT_, which is expressed as a diameter of a semicircle at low frequencies. Figure 3B shows the Nyquist plots for each step of the fabrication process of the electrode. The bare Ag electrode (curve a) shows an exceedingly small semicircle (R_ct_ = 440.75 Ω), because of the high conductivity of Ag. With the immobilization of 11-MUA on top of Ag WE, the semicircular diameter increased (R_ct_ = 590.81 Ω), revealing the blocking of electron transfer (curve b). In the next stages (curve c, d and e), the semicircle increased progressively, proving the hindrance of the electron transfer rate of EDC/NHS (R_ct_ = 992.86 Ω), aptamer (R_ct_ = 1066.40 Ω), and BSA (R_ct_ = 1254.70 Ω). This result demonstrated a proper agreement with the result of CV and proved the effectiveness of the fabrication steps performed.

To investigate the nature of the electrochemical reaction of the screen-printed Ag electrode and aptamer-immobilized screen-printed Ag electrode, the effect of scan rate from 20 to 200 mV/s was measured (Figures S1 and S2). The cathodic peak current in both cases increased linearly (Figures S3 and S4) with the increment of the scan rate suggesting that diffusion-controlled processes take place. Using the Randles–Sevcik equation [40], the electroactive surface area of both sensors was measured and found to be 0.59 and 0.34 cm^2^, respectively, showing that the immobilization of the aptamer induced a decrease in the effective surface area.

### 3.2. Working Principle of the Aptasensor

**Figure 4:**
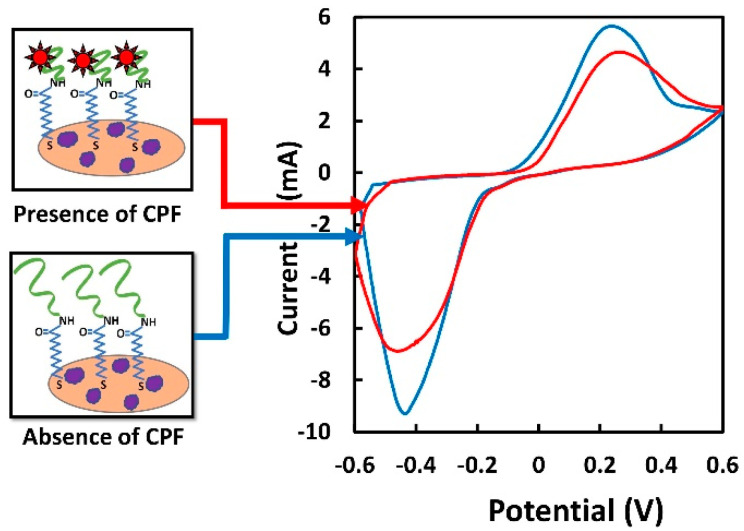
CV analysis without the presence of CPF (blank solution containing 0.01 M PBS) (blue) and with the presence of 100 ng/mL CPF in 0.01 M PBS solution (red). Screen-printed Ag/AgCl was used as the RE.

Figure 4 illustrates the working principle of the aptasensor reported in this paper, where the blue and red CV were generated with the absence and presence of CPF, respectively. In the absence of CPF, the immobilized aptamers were distant from the electrode surface; hence, a high current was generated [59]. In contrast, in the presence of CPF, the latter bound to the aptamer molecules, and as a result, the aptamers underwent a conformational change and bent closer to the electrode surface as previously reported [60,61]. In this manner, the aptamers created a barrier toward the electron transfer, and thereby, the current generated was reduced.

### 3.3. Optimization of the Experimental Conditions

**Figure 5:**
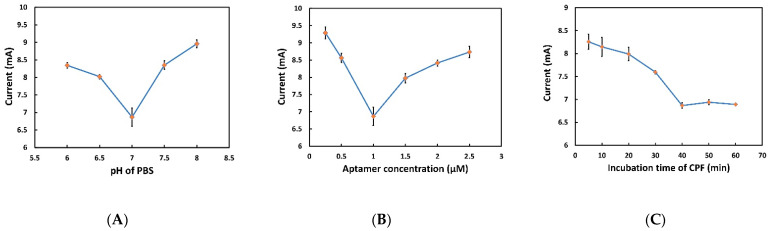
(**A**) Optimization of pH of PBS for the aptasensor characterization. The reduction peak current (absolute value) obtained from the aptasensor through CV at different pH of PBS (6, 6.5, 7, 7.5, and 8). (**B**) Optimization of aptamer concentration for the aptasensor characterization. The reduction peak current (absolute value) obtained from the aptasensor through the CV with different aptamer concentrations (0.25, 0.5, 1, 1.5, and 2 µM). (**C**) Optimization of CPF incubation time for the aptasensor characterization. The reduction peak current (absolute value) obtained from the aptasensor through CV achieved by different incubation times (5, 10, 20, 30, 40, 50, and 60 min) of CPF.

To achieve the maximum efficiency and sensitivity of the biosensor, optimization of the experimental parameters such as pH of PBS, aptamer concentration, and incubation time of CPF are of uttermost importance. Since pH has a crucial effect on the redox reaction and the overall performance of the aptamer, it needs to be optimized in the PBS solution (0.01 M) used for preparing the analyte. It was also observed from Guo et al. [62] that the pH of PBS can alter the affinity between the aptamer and the analyte. To find out the optimum pH level of PBS, the sensor response was recorded through CV using various levels of pH from 6 to 8. Figure 5A shows the current generated as a function of different pH values of electrolytes. While increasing the pH value from 6.0 to 7.0, the lowest reduction peak current (6.86 ± 0.26 mA) was achieved at a specific pH value (pH 7.0). Increasing the pH value further from 7.0 to 8.5 resulted in an increase in current. As mentioned earlier, the aptamers used in this work are single-stranded DNA molecules, which consist of a sequence of nucleotides. Briefly, the most important force linking each single-stranded (ss) DNA and forming double-stranded (ds) DNA that gives the aptamers their secondary structure and gives it the high affinity toward the target is the hydrogenic bond formed between cytidine (C) and guanosine (G), and adenosine (A) and thymidine (T). Hydrogen bond formation is controlled by pH; for example, if the electrolyte pH is lower than the pH of the aptamer solution, the H^+^ present in the surrounding environment of dsDNA is high, and therefore the hydrogen bonds between C-G and A-T may break in a competitive manner, thereby resulting in a different aptamer arrangement and decreasing the affinity of the aptamer. In contrast, a high pH electrolyte contains an abundance of hydroxide ions (negatively charged), which can disrupt the hydrogen bonding that gives aptamers their structure. This is obtained by pulling the hydrogen ions from the base pairs. Thus, if the pH of the electrolyte is less or more than the pH used in the aptamer isolation process, i.e., SELEX, the aptamer will be induced to undergo a change in its secondary structure, which will eventually result in a decrease in affinity with the target. To conclude, to obtain the best performance from the aptamers, the same pH used during the SELEX process should also be used for the electrolyte; thereby, a pH = 7 was chosen for the entire voltammetry experiment [63].

Next, the effect of the surface density of the aptamers on the WE, on the performance of the aptasensor, was investigated. A wide range of aptamer concentrations was used. From Figure 5B, it can be observed that the reduction peak current reaches the minimum value while increasing the concentration from 0.25 to 1 µM, and then it starts to increase while increasing the concentration from 1 to 2.5 µM. Alternatively, immobilizing the aptamers solution with less than 1 µM creates a low aptamer concentration, which may not be enough for the conformational changes with the presence of the analyte and which may not be allowed to pass through the electrons followed by a higher reduction current. The concentration of aptamer higher than 1 µM may lead to intermolecular hybridization among the aptamers, which consequently interferes with the conformational change of the aptamers on which the aptasensor working principle is based. Therefore, most of the aptamers will stay distant from the working electrode in the presence of CPF, which results in a higher reduction peak current. This result was in agreement with the previous work by Fu et al. [19]. Accordingly, 1 µM aptamer concentration was chosen for subsequent assays.

The incubation time of CPF is another parameter that needs to be optimized because the aptamer needs time to bind successfully with the target analyte. To find out the optimum incubation time, a wide range of incubation times of CPF from 5 to 60 min was used. As shown in Figure 5C, the reduction peak current decreased when incubation time increased from 5 to 40 min. After 40 min, the current was stable, proving that the specific binding between aptamers and CPF reached saturation [28]. Therefore, the optimum incubation time for CPF was selected as 40 min for the subsequent experiments. Each experiment was carried out 5 times, and the average value with error bar is shown in Figure 5.

### 3.4. Analytical Performance of the Aptasensor

**Figure 6:**
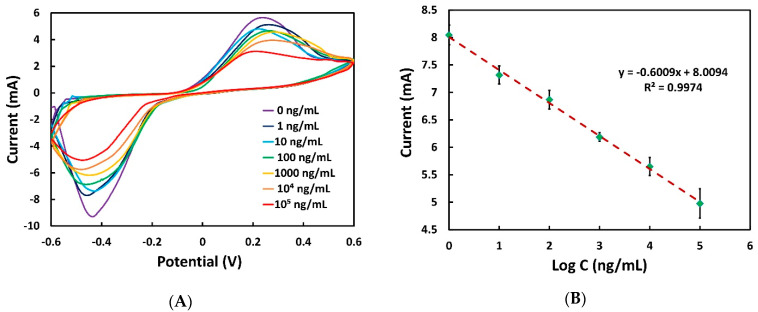
(**A**) CV at different concentrations of CPF (0, 10^0^, 10^1^, 10^2^, 10^3^, 10^4^, 10^5^ ng/mL) in 0.01 M PBS. (**B**) Calibration plot of aptasensor for CPF detection, where the highest reduction peak current is shown in absolute value versus CPF concentration. Here, 1 µM of aptamer was used as previously optimized, and the optimized incubation time of CPF was set as 40 min. The average reduction peak current was obtained from three sensors where SD with error bar is shown.

Considering all the optimal experimental conditions, i.e., 1 µM of aptamer, pH 7.0 of PBS and 40 min of CPF optimization time, the sensitivity of the aptasensor was investigated for CPF detection ranging from 1 to 10^5^ ng/mL. It is shown from Figure 6A that with the increment of the concentration of CPF, the reduction peak current decreased proportionally. The more that the amount of CPF is captured by the working surface of the biosensor, the higher the number of aptamers that undergo a conformational change (bending closer to the surface of the working electrode), thereby increasing the electron transfer resistance. The relationship between the reduction peak current and the logarithm of target concentrations is plotted in Figure 6B, and a strong linear relationship was found with the regression equation of the calibration curve y = 0.6009 mA/ng x − 8.0094 mA (R^2^ = 0.9974). In addition, the limit of detection was also calculated using Equation (1) and was found to be 0.097 ng/mL

LOD = (3.3 STDEV I_o_)/m (1)

where I_o_ is the generated peak current of blank (0 ng) concentration of CPF, and m is the slope of the linear response curve.

The performance of this biosensor was compared with recently developed aptasensors for CPF detection as shown in Table 1. Until now, sensors for CPF detection are mainly based on GCE and pencil graphite electrodes (PGE) functionalized with different nanomaterials to form a complex and multilayer structure. Conversely, here we propose an aptasensor realized in a quite simple manner using cost-effective screen-printed Ag electrodes that have the potential to compete with other aptasensors in terms of linear detection range and LOD (see Table 1).

### 3.5. Selectivity, Repeatability, and Stability of the Aptasensor

**Figure 7:**
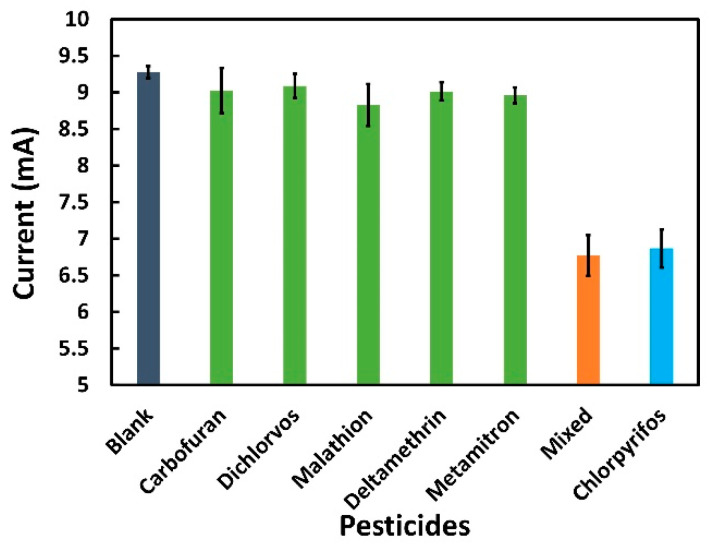
Selectivity test of aptasensor where the average reduction peak current (absolute value) of different pesticides (carbofuran, dichlorvos, malathion, deltamethrin, metamitron, chlorpyrifos, and mixture of all) were obtained through the CV.

Selectivity plays a particularly key role in investigating the reliability of aptasensors for complex analytes such as CPF. To assess the selectivity of the proposed biosensor, five commonly used pesticides (carbofuran, dichlorvos, malathion, deltamethrin, and metamitron) were tested. First, 100 ng/mL of each pesticide was prepared in 0.01 M PBS and measured in terms of reduction peak current by CV. The generated current was then compared with the blank solution (0.01 M PBS). As shown in Figure 7, there were no changes between the current signal generated from the other pesticides and the blank concentration, proving the absence of CPF. In addition, all pesticides including CPF (100 ng/mL each) were mixed together, each containing 100 ng/mL and compared with a solution containing only CPF. As shown in Figure 7, CPF and the mixture of all pesticides showed the current in the same range, which proves that the sensor has no co-interfering from the presence of other analytes and is able to detect CPF in a complex environment. Error bars from standard deviation for all experiments were in a considerable range (maximum SD 0.31 mA), indicating the high selectivity of the aptasensor for CPF detection.

For the repeatability test, each aptasensor was assessed by five consecutive measurements with the same conditions, and the reduction peak current amplitude of each repetition was compared to the first performed. After testing each time, the sensor was rinsed gently with DI water, dried with natural air and prepared for the next test. As shown in Figure S5, the sensor is usable up to a fourth time (4.67% changes in reduction peak current compared to first test). Gradual degradation of sensor performance was observed. The continuous application of potential can degrade the performance of the pseudo-reference electrode [66]. However, it is not a major concern because the sensor was meant to be a one-time disposable device.

**Figure 8:**
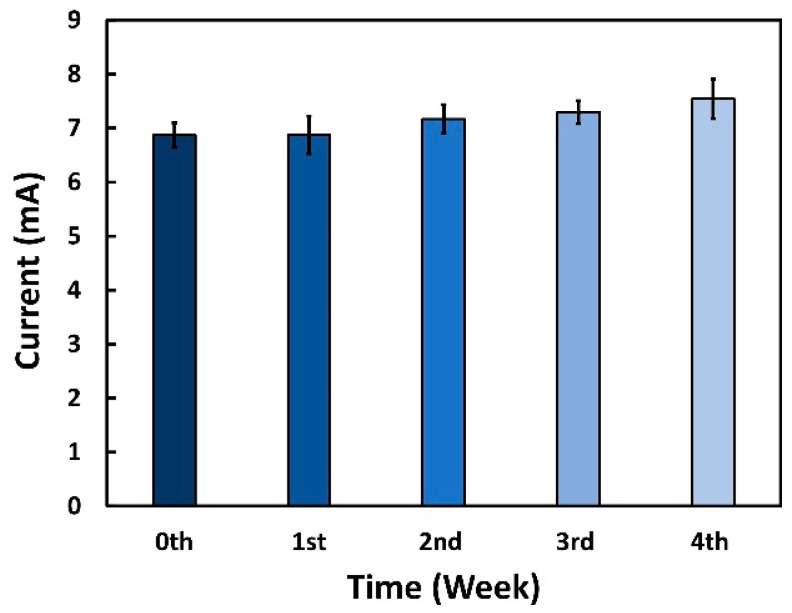
Stability over time for the aptasensor until 1 month. Here, the average reduction peak current shown in the absolute value of the aptasensor through CV was obtained every week.

The long-time stability of the aptasensor is a key factor to determine the possible measurement drifts because of aging effects. For this experiment, all the biosensors were prepared on the same day and kept in the refrigerator at 4 °C for further use. The stability over time was checked every week for one month. Each week, five aptasensors were used to detect 100 ng/mL of CPF through CV measurements starting from day zero, and the results of the progressive weeks were compared with day zero ones. The results in Figure 8 demonstrate that the reduction peak currents generated from the aptasensor were similar after a week (coefficient of variance 3.28%); however, it started to deviate after the 2nd week (coefficient of variance 5.06%) because of the degradation of the aptamer. The changes in reduction peak current were 0.10%, 4.38%, 6.23%, and 9.82% from the 1st to 4th week, respectively, compared to the zeroth day sensor indicating that the aptasensor was in a perfectly working condition up to the 2nd week.

### 3.6. Real Sample Analysis

The aptasensor was evaluated on a real sample using a standard addition method to determine its practical usage [67]. The experiments were conducted using the previously optimized condition, and the results are presented in Table 2. The recovery rate after adding 100 and 1000 ng/mL in the grape sample was 105.7% and 97.7%, and in the banana, the samples were 103.5% and 104.1%, respectively. The coefficient of variance was calculated and found to be 2.77% to 4.55% for the banana and 1.75% to 3.78% for the grape sample. This indicates that the aptasensor has the high accuracy and reliability to detect CPF in real samples.

## 4. Conclusions

In conclusion, this paper proposes an extremely sensitive and easy-to-fabricate aptasensor for the detection of CPF. First, to enhance the performance of the aptasensor, different parameters have been optimized, including the pH of PBS, the concentration of aptamers, and the incubation time of CPF. In optimum conditions, this aptasensor exhibits outstanding performance with a linear detection range of 1 to 10^5^ ng/mL and a limit of detection of 0.097 ng/mL. Furthermore, the selectivity of this aptasensor was investigated, and it was found that other pesticides have a negligible effect on the detection of CPF. Stability over time is another important parameter that was evaluated. The aptasensors were kept in a refrigerator at 4 °C for one month, where they showed good stability during the first 14 days, but then the sensitivity decreased due to degradation of the aptamer. Finally, the aptasensor was challenged with real samples (grape juice and banana extract) with high recovery rates. Considering all the results, we can conclude that this versatile, easy to construct, and cost-effective aptasensor has enormous potential in the detection of CPFs. Further development of this aptasensor can be realized in terms of stability over time and portability by realizing a custom-designed portable readout system that will allow for on-site analysis in the future.

## Figures and Tables

**Table 1:** Comparison of different aptasensor for the determination of CPF.

Fabrication of Electrode	Methods	Linear Detection Range (ng/mL)	Limit of Detection (ng/mL)	References
SWCNT on GCE	DPV	0.1–150	0.07	[30]
MWCNT on GCE	CV	1–10^5^	0.33	[28]
AgNP on GCE	Colorimetry	20–300	7.4	[64]
MIP on PGE	EIS	20–300	4.5	[65]
AgNP on GCE	Colorimetry	70–1750	0.35	[17]
SPAgE	CV	1–10^5^	0.097	This work

SWCNT: Single-walled carbon nanotubes; GCE: glassy carbon electrode; MWCNT: multi-walled carbon nanotubes; DPV: differential pulse voltammetry; CV: cyclic voltammetry; EIS: electrochemical impedance spectroscopy; AgNP: silver nanoparticles; MIP: molecularly imprinted polymers; PGE: pencil graphite electrode.

**Table 2:** CPF detection in different fruit samples by the aptasensor.

Sample	Added CPF (ng/mL)	Detected by the Sensor (ng/mL)	Relative Recovery (%)
Grape	0	-	-
	100	105.77 ± 4.73	105.77
	1000	976.92 ± 27.11	97.69
Banana	0	-	-
	100	104.06 ± 3.94	104.06
	1000	1035.73 ± 18.14	103.57

## Data Availability

Not applicable.

## Supplementary Materials

The following supporting information can be downloaded at: https://www.mdpi.com/article/10.3390/s22072754/s1. Figure S1: Cyclic voltammogram performed on the Ag electrode where the electrolyte is 5 mM of [Fe(CN)_6_]^3−/4−^ in 0.1 M KCl at various scan rates (20 to 200 mV/s); Figure S2: Cyclic voltammogram performed on the aptamer immobilized Ag electrode where the electrolyte is 5 mM of [Fe(CN)_6_]^3−/4−^ in 0.1 M KCl at various scan rates (20 to 200 mV/s); Figure S3: Linear relationship of peak current versus square root of the scan rate of the Ag electrode; Figure S4: Linear relationship of peak current versus square root of the scan rate of the aptamer immobilized Ag electrode; Figure S5: Repeatability test of the aptasensor. The reduction peak current obtained by CV was taken from same sample repeating 5 times measurement. The percentage is the value of the degradation of the electrode compared to the first one. The experiments were performed in triplicate and the error bars from the standard deviations are shown.
