# Supplementary material for: An Aptasensor Based on a Flexible Screen-Printed Silver Electrode for the Rapid Detection of Chlorpyrifos
Source: Sensors (Basel). 2022 Apr 2;22(7):2754. doi: 10.3390/s22072754 (PMC9003324; doi:10.3390/s22072754)
Supplement: Supplementary file 1 [file sensors-22-02754-s001.zip › sensors-1646097-supplementary.pdf]

Supplementary Materials

An aptasensor based on a flexible screen-printed silver electrode for the rapid detection of chlorpyrifos

AKM Sarwar Inam [1,2], Martina Aurora Costa Angeli [1*], Ali Douaki [1], Bajramshahe Shkodra [1], Paolo Lugli [1], and Luisa Petti [1]

[1] Sensing Technologies Laboratory, Faculty of Science and Technology, Free University of Bozen-Bolzano, Bolzano, 39100, Italy
[2] Department of Nutrition and Food Engineering, Daffodil International University, Dhaka 1207, Bangladesh
* Correspondence: MartinaAurora.CostaAngeli@unibz.it

1. Electrochemical characterization

The effect of scan rate from 20 to 200 mV/s was investigated to understand the nature of the electrochemical reaction of screen-printed Ag electrode and aptamer immobilized screen-printed Ag electrode (Figure S1 & S2).

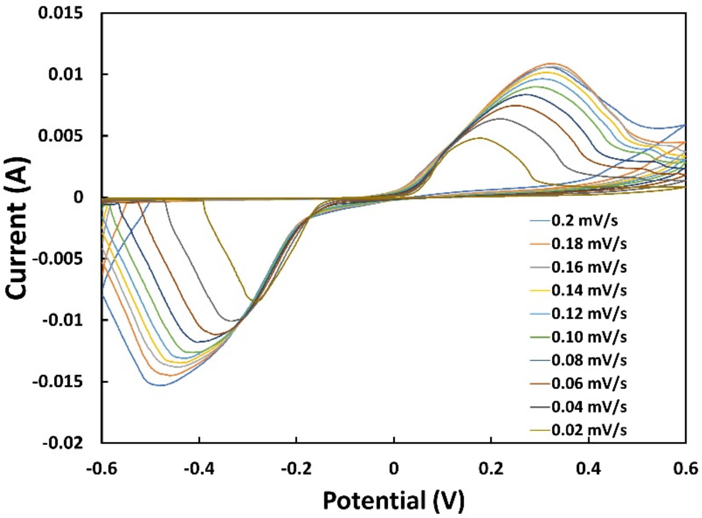

Figure S1: Cyclic voltammogram performed on the Ag electrode where the electrolyte is 5 mM of $[Fe(CN)_6]^{3-/4-}$ in 0.1 M KCl at various scan rates (20 to 200 mV/s).

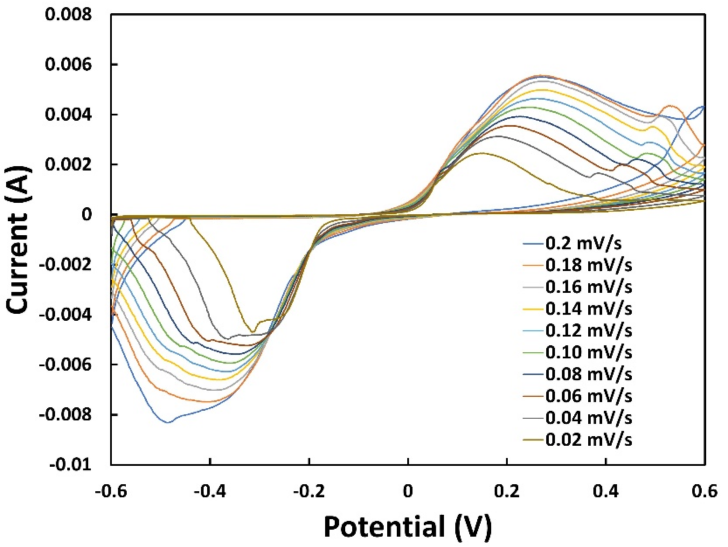

Figure S2: Cyclic voltammogram performed on the aptamer immobilized Ag electrode where the electrolyte is 5 mM of $[Fe(CN)_6]^{3-/4-}$ in 0.1 M KCl at various scan rates (20 to 200 mV/s).

The cathodic peak current in both cases increased linearly (Figure S3 & S4) with the increment of the scan rate suggesting that diffusion-controlled processes take place.

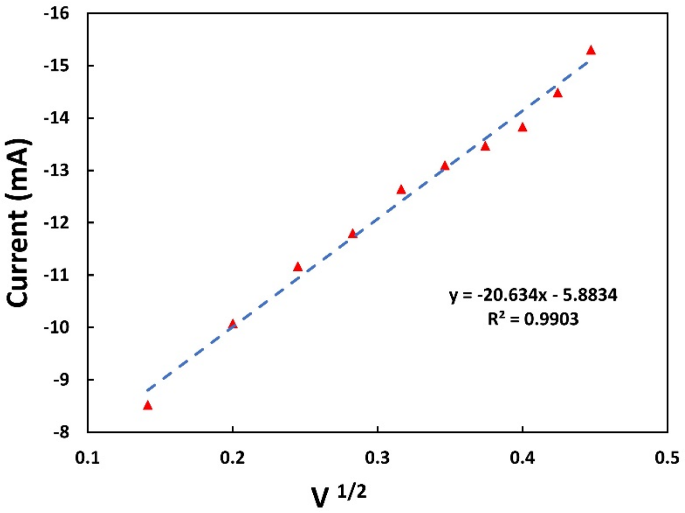

Figure S3: Linear relationship of peak current versus square root of the scan rate of the Ag electrode.

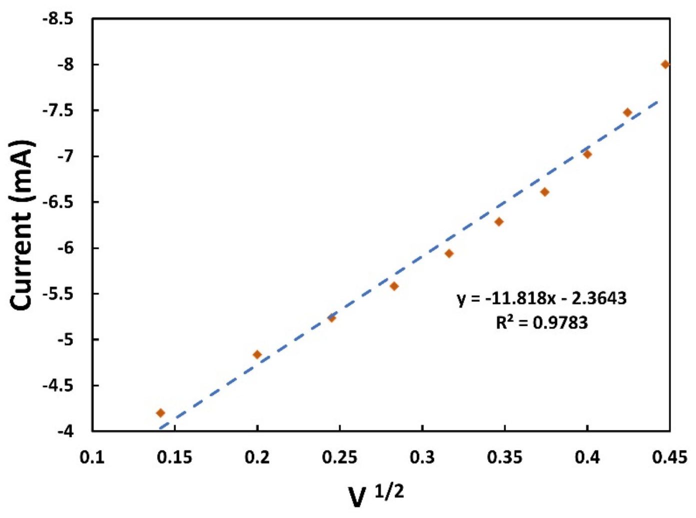

Figure S4: Linear relationship of peak current versus square root of the scan rate of the aptamer immobilized Ag electrode.

2. Repeatability test of the sensor

For the repeatability test, the same sensor was tested 5 consecutive times and the reduction peak current was taken in Figure S5. Each time after examining, the sensor was rinsed with DI water and dried with ambient air and kept ready for the next test.

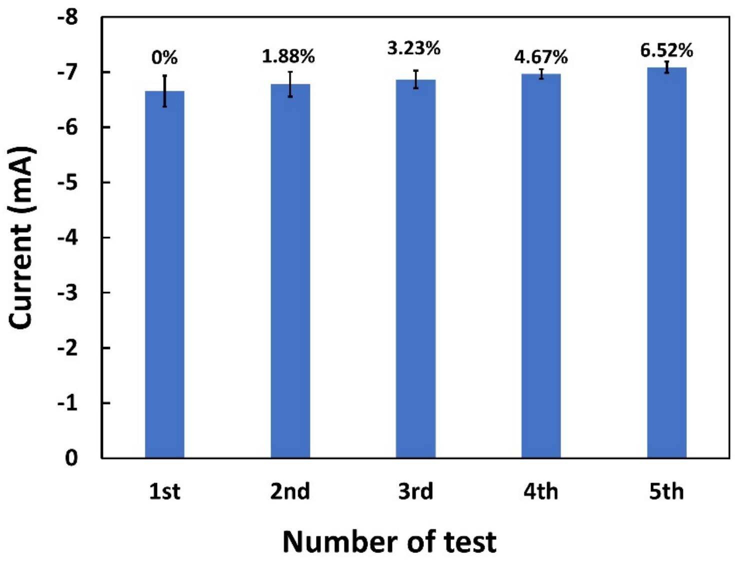

Figure S5: Repeatability test of the aptasensor. The reduction peak current obtained by CV was taken from same sample repeating 5 times measurement. The percentage is the value of the degradation of the electrode compared to the first one. The experiments were performed in triplicate and the error bars from the standard deviations are shown.
